# Assessing Facial Skin Hydration via Tissue Dielectric Constant (TDC): Suitability of Single Versus Multiple Measurements

**DOI:** 10.7759/cureus.110902

**Published:** 2026-06-15

**Authors:** Harvey N Mayrovitz

**Affiliations:** 1 Medical Education, Nova Southeastern University Dr. Kiran C. Patel College of Allopathic Medicine, Davie, USA

**Keywords:** dermal water, eczema, facial skin, psoriasis, rosacea, skin hydration, skin water, tdc, tissue dielectric constant

## Abstract

Background: Noninvasive tissue dielectric constant (TDC) measurements provide a useful index of local skin water because TDC, measured at 300 MHz, depends directly on the amount of free and bound water in the measurement volume. Since the hydration status of facial skin is a fundamental factor affecting multiple dermatological conditions, including rosacea, psoriasis, and eczema, TDC values may be useful for assessing dermal hydration in these conditions. Although some data on female facial values have been reported, data on male facial TDC are quite limited. Further, since multiple skin sites often need to be evaluated, time constraints in the clinic may limit the acquisition of useful data if multiple measurements are required at each site. Thus, the study's goal was to determine if a single TDC measurement, rather than averaging multiple measurements, would be sufficient.

Methods: TDC was measured in triplicate bilaterally at the forehead (F), cheek (C), and jowl (J) areas to an effective depth of 1.5 mm in 40 male subjects while they were supine and then seated after 15 minutes at each position. The first TDC value (TDC1) was compared with the average of the first and second (TDC12) and with the triplicate average (TDC123).

Results: Group-average TDC values did not differ statistically among TDC1, TDC12, and TDC123 at any facial site, whether measured with the subject supine or seated. Supine TDC1 values (mean ± SD) for the F, C, and J areas were 42.11 ± 3.19, 39.15 ± 5.49, and 43.44 ± 4.56, respectively. The corresponding TDC values with subjects seated were 40.68 ± 2.99, 38.49 ± 5.54, and 41.97 ± 4.37. Supine-seated differences were statistically significant at the forehead and jowl areas (p < 0.001) but not at the C (p = 0.193). TDC differences among sites, though small, were statistically significant (p < 0.001) at both positions. C had the lowest TDC value. On an individual basis, no subject had a TDC1 value that differed by more than 3% from the triplicate average.

Conclusions: Results indicate that when clinical time is a concern, or when experimental time needs to be shortened for any reason, accurate facial TDC data may be obtained at the F, C, and J areas using a single TDC measurement with the subject either supine or sitting, whichever is appropriate for the patient and examiner.

## Introduction

Tissue dielectric constant (TDC) measurements provide a noninvasive, rapid way of assessing skin water content locally [1]. This is possible because the TDC value is directly dependent on the free and bound water within the measured tissue volume [2-5]. Such measurements have been used to evaluate and track skin water from a physiological standpoint [6-10] and in a variety of clinical conditions, including lymphedema [11-15], diabetes [16,17], and other conditions. Each skin measurement requires touching the skin with the sensor of a handheld probe (refs) for up to 10 seconds to obtain a reading, and most studies have used the average of triplicate measurements to assess the value at the measurement site. However, when measurements are to be made in a busy clinic, the requirement for three vs. one measurement per site can be a significant impediment. To determine the differential utility of a single measurement versus the average of multiple measurements, a study was conducted using TDC values obtained from the upper and lower extremities of healthy participants [18]. It was found that although one measurement was suitable for the upper extremities, it was not suitable for the lower extremities, indicating differences between anatomical regions. No such investigation has been undertaken into other anatomical regions. An area of current interest is the face, especially since skin hydration status is implicated in conditions such as rosacea [19,20], psoriasis [21,22], and eczema [23,24]. Thus, the aim of this study was to evaluate and compare facial skin TDC values obtained from single, duplicate, and triplicate sequential measurements across multiple bilateral facial areas. 

## Materials and methods

Subjects

A total of 40 male subjects were recruited from first- and second-year medical students, faculty, and staff at Nova Southeastern University, Davie, FL, USA, where this study was conducted from August 13, 2009, through May 4, 2013. Participants were evaluated during a single session after the nature of the study was explained, and each signed an approved university institutional review board consent form (approval number: NSU 07300901F). Inclusion criteria were males aged between 21 and 35 years with no facial hair or beard, no facial sores, or visibly apparent facial skin condition. Subjects were instructed to shave at least three hours prior to their scheduled appointment and were excluded from participation if they arrived at their scheduled appointment with significant facial hair. The group's age (mean ± SD) was 25.35 ± 2.62 years, and their body mass index (BMI) was 25.81 ± 5.37 kg/m². Considering standard BMI classifications, seven subjects were obese, 14 were overweight, 18 were normal weight, and one was underweight.

Measurement method

TDC was measured using a handheld probe connected to a control box (MoistureMeterD, Delfin Technologies, Kuopio, Finland). In use, the 20 mm-diameter probe surface is held in contact with the skin, and within 10 seconds, the TDC value is determined and displayed on the control box. The probe used measures to an approximate effective depth of 1.5 mm, defined as the depth at which the incident electric field falls to 1/e of its skin-surface value. The measured quantity, TDC, is also called relative permittivity, the ratio of tissue permittivity to free-space permittivity, and is thus dimensionless. For reference, the dielectric constant of pure distilled water at a temperature of 32°C is about 76. The probe acts as a coaxial transmission line, through which a 300-MHz signal is transmitted. Reflections depend on the tissue’s complex permittivity, which in turn depends on the signal frequency and the TDC (the real part of the complex permittivity). At the frequency used, the contribution of conductivity to permittivity is small, so TDC is mainly determined by water molecules (free and bound). Thus, the device measures and analyzes the dielectric constant, which is proportional to tissue water content.

Face measurement sites

**Figure 1:**
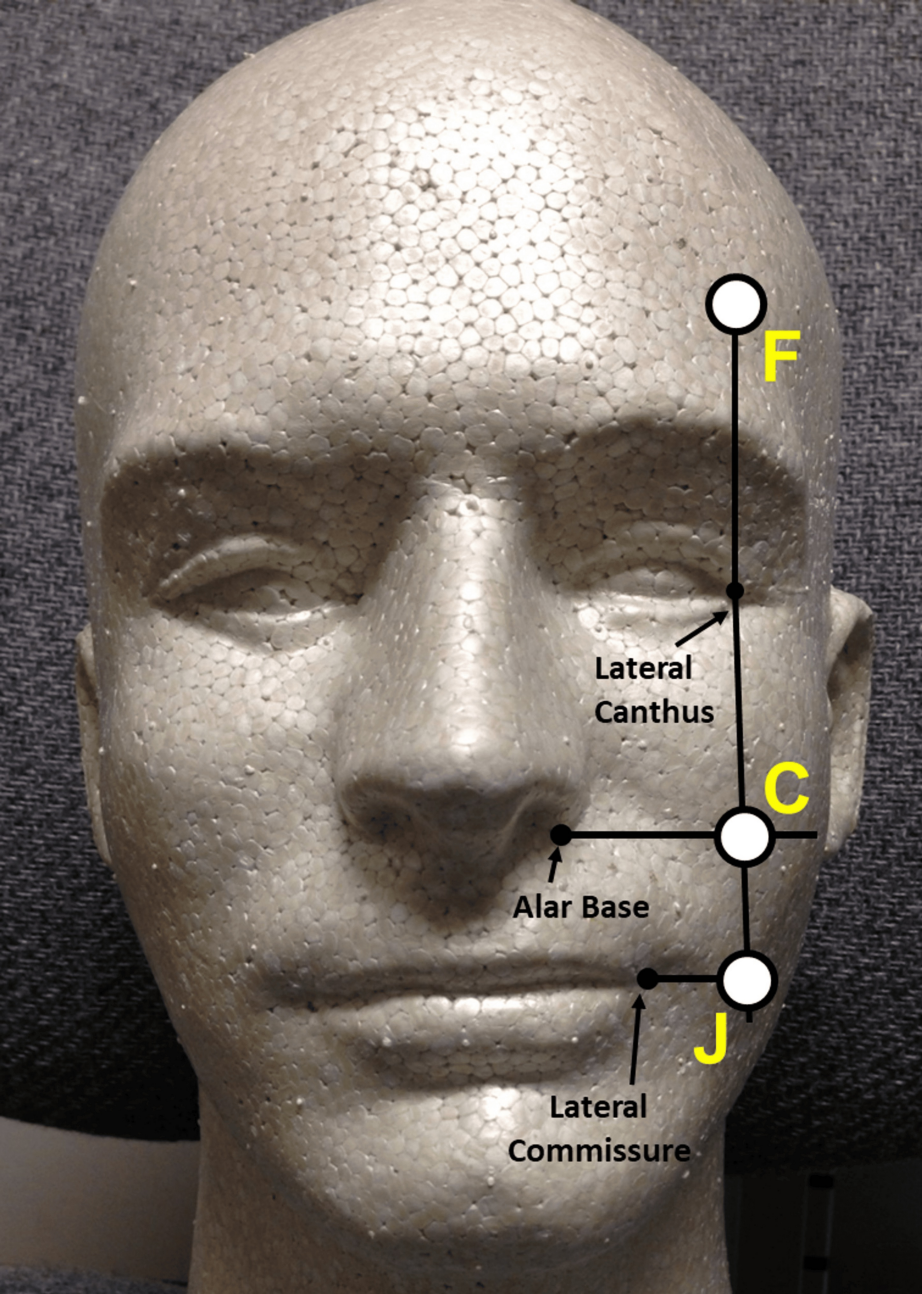
Tissue dielectric constant (TDC) facial measuring sites TDC was measured on the left and right face sides along a vertical line adjacent to the lateral canthus of the eye. This is shown on the left side of the face in this figure. The forehead site (F) was 6 cm caudal to the lateral canthus. The cheek site (C) was determined by the intersection of the vertical line with a perpendicular from the nose alar base. The jowl site (J) was determined by the intersection of the vertical line with a perpendicular from the lateral commissure. This figure was created by the author by photographing a model image and then importing it into Microsoft PowerPoint (Microsoft Corp., Redmond, WA, USA) and annotating it as shown.

Measurements were taken on the left and right sides of the face, along a vertical line adjacent to the lateral canthus of the eye, as shown in Figure 1 for the left side. The forehead site (F) was 6 cm caudal to the lateral canthus. The cheek site (C) was determined by the intersection of the vertical line with a perpendicular line starting from the nose alar base. Jowl site (J) was determined by the intersection of the vertical line with a perpendicular line starting from the lateral commissure.

Measurement sequence

With the subject supine, left-side sites were marked, followed by marking of the right side. The supine measurement sequence started after the subject had been supine for 15 minutes. TDC was measured first on the left side, starting with F, then progressing to C, and finally to J. This sequence was repeated on the right side. This pattern was repeated twice more to obtain triplicate measurements at each of the six sites. 

Analysis

For analysis, the first TDC measurement at a site was defined as TDC1. The average of the first and second measurements was defined as TDC12. The average of all three measurements was defined as TDC123. TDC values from the left and right sides of the face were combined to yield 80 TDC value sets for the F, C, and J sites. The distribution of TDC values among subjects at each site was tested for normality using the Shapiro-Wilk test, with a significance level > 0.05 indicating that the null hypothesis of normality was not rejected. Tests for overall differences among TDC1, TDC12, and TDC123 were conducted at each site individually using a general linear model (GLM, SPSS version 13 (SPSS Inc., Chicago, IL, USA)) for repeated measures and applied to differences among TDC-determined values and among sites. Differences between supine and seated TDC values were tested for using paired t-tests. No sample size calculation was done for this study design. 

## Results

Normality assessment

TDC values at F were normally distributed with the subject supine and seated, with Shapiro-Wilk test significance ranging from 0.422 to 0.521 while supine and from 0.354 to 0.720 while seated. In contrast, TDC values at the C and J areas deviate from a normal distribution in both supine and seated positions, with significance ranging from 0.001 to 0.007.

TDC values by facial site and TDC determination with subjects supine

Table 1 summarizes the mean TDC values for all 40 subjects by facial location while they were supine, using the TDC1 measurement, the average of TDC12 measurements, and the triplicate average of the three consecutive measurements. Based on the GLM repeated-measures model, there was no statistically significant difference among these TDC values across the F, C, or J areas. However, tests for differences among sites show a significant overall difference (p = 0.001), with TDC values at C lower than at the other two sites (p = 0.001). This difference was present whether TDC values for the site were determined using only the first TDC measurement, the average of the first and second, or the triplicate average.

**Table 1:** Tissue dielectric constant (TDC) values by facial site with the subject supine for 40 participants Table entries are the mean ± standard deviation for TDC values at each facial site. TDC1, TDC12, and TDC123 correspond to the TDC value from the first measurement, the average of the first and second measurements, and the triplicate average, respectively.  The significance values are for the overall difference among TDC measurements based on a general linear model for repeated measures with TDC values as the repeated measure. The p-values are for the overall difference among sites based on a general linear model for repeated measures with site as the repeated measure. All tests were done for 40 participants.

Site	TDC1	TDC12	TDC123	Significance
Forehead	42.11 ± 3.19	41.99 ± 3.03	41.88 ± 2.99	0.093
Cheek	39.15 ± 5.49	39.16 ± 5.34	39.22 ± 5.10	0.789
Jowl	43.44 ± 4.56	43.24 ± 4.41	43.18 ± 4.31	0.208
p-value	< 0.001	< 0.001	< 0.001	

**Figure 2:**
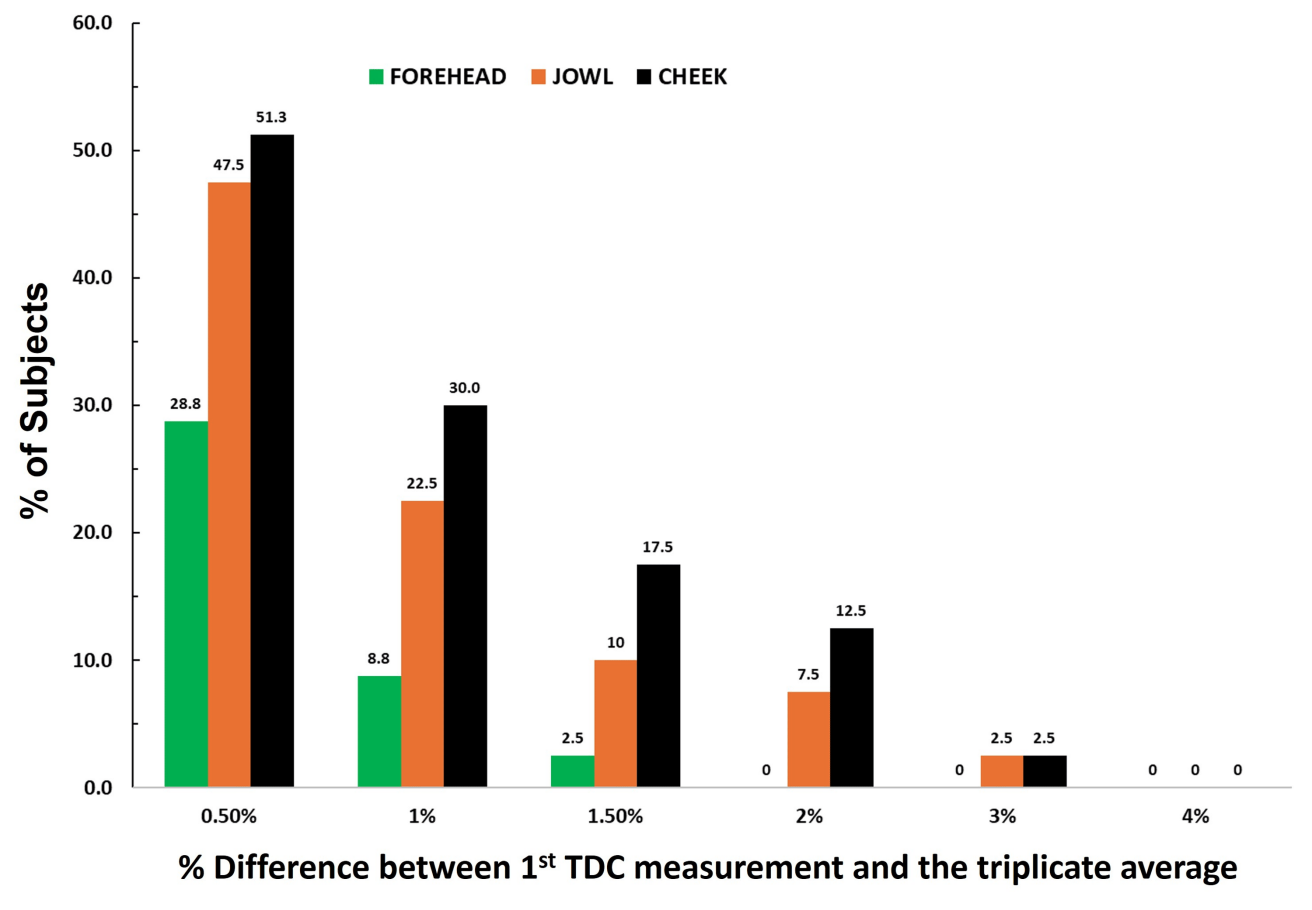
Differences between the first and triplicate tissue dielectric constant (TDC) measurements with subjects supine for 40 participants This figure shows the number of subjects for whom the TDC measurements exceed a specified percentage difference between the first TDC measurement and the triplicate average at each site.

A more granular examination of the differences obtained on an individual subject basis is presented in Figure 2. This figure shows the number of subjects for whom the measurements exceed a specified percentage difference between the TDC1 measurement and the triplicate average at each site. For F, no subject showed a difference between these TDC values that exceeded 3%. For the C and J areas, no difference exceeded 4%.

TDC values by facial site and TDC determination with subjects sitting

Table 2 summarizes the mean TDC values for all 40 subjects by facial location while they were sitting, using the first TDC measurement, the average of the first and second measurements, and the triplicate average of the three consecutive measurements. Based on the GLM repeated-measures model, there was no statistically significant difference among these TDC values across F, C, or J areas. However, tests for differences among sites show a significant overall difference (p = 0.001), with TDC values at C lower than at the other two sites (p = 0.001). This difference was present whether TDC values for the site were determined using only the TDC1 measurement, the average of the first and second, or the triplicate average. These patterns are essentially the same as those observed with the subject supine. However, all seated values at the corresponding facial sites are slightly, but statistically, lower than when subjects were supine. 

**Table 2:** tissue dielectric constant (TDC) values by facial site with the subject sitting for 40 participants Table entries are the mean ± standard deviation for TDC values at each facial site with the subject sitting. TDC1, TDC12, and TDC123 correspond to the TDC value from the first measurement, the average of the first and second measurements, and the triplicate average, respectively. The significance values are for the overall difference among TDC measurements based on a general linear model for repeated measures with TDC values as the repeated measure. The p-values are for the overall difference among sites based on a general linear model for repeated measures with site as the repeated measure. All tests were done for 40 participants.

Site	TDC1	TDC12	TDC123	Significance
Forehead	40.68 ± 2.99	40.66 ± 2.62	40.71 ± 2.43	0.957
Cheek	38.49 ± 5.54	38.58 ± 5.10	38.61 ± 4.98	0.778
Jowl	41.97 ± 4.37	42.29 ± 4.14	42.25 ± 4.05	0.137
p-value	< 0.001	< 0.001	< 0.001	

**Figure 3:**
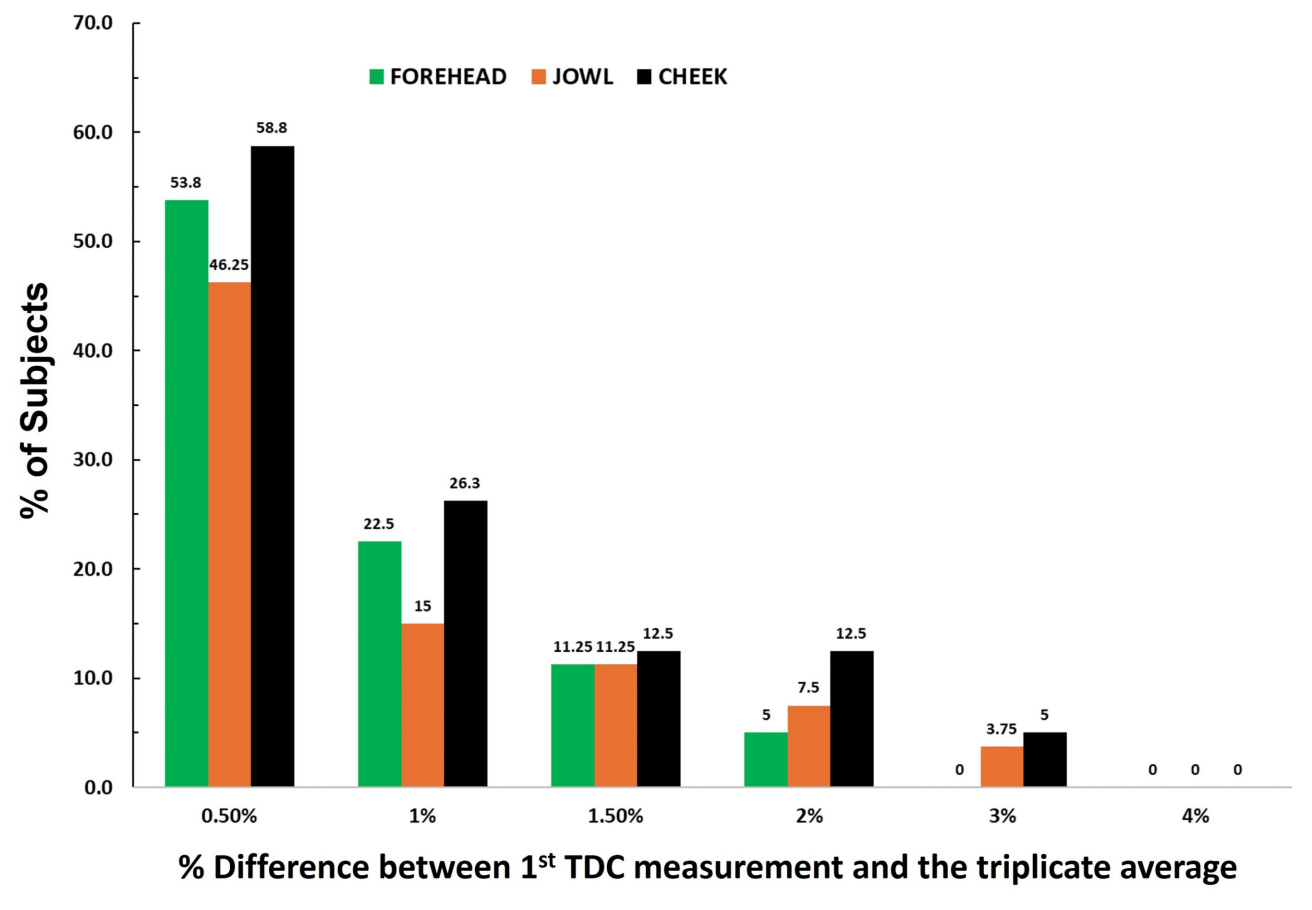
Differences between first and triplicate tissue dielectric constant (TDC) measurements with subjects seated for 40 participants This figure shows the number of subjects for whom the measurements exceed a specified percentage difference between the first TDC measurement and the triplicate average at each site.

As done with the supine position data, a more granular examination of the differences obtained on an individual subject basis for subjects in the seated position is presented in Figure 3. This figure also shows the number of subjects for whom the measurements exceed a specified percentage difference between the TDC1 and the triplicate average at each site. Similar to the findings when subjects were supine, no subject showed a difference in these TDC values at the forehead exceeding 3%. For the C and J areas, no difference exceeded 4%.

Differences in TDC values between subject positions

Table 3 summarizes the effect of the subject position on the first TDC measurement and on the triplicate average at each site. At all three facial sites, the TDC values obtained with subjects in a seated position tended to be lower than those obtained with subjects supine. The difference was statistically significant in the F and J areas but not in the C area. However, whether for the first or triplicate average TDC measurement, the average percentage difference between measurements with the subject supine and sitting was less than 1% for all sites.

**Table 3:** Tissue dielectric constant (TDC) differences between positions for 40 participants Table entries are the mean ± SD of TDC values for the first and triplicate averages. All p-values were calculated using a paired t-test comparing supine and sitting TDC values for all 40 participants.

	First TDC Measurement			Triplicate Average TDC Value		
Site	Supine	Sitting	p-value	Supine	Sitting	p-value
Forehead	42.11 ± 3.19	40.68 ± 2.99	< 0.001	41.88 ± 2.98	40.71± 2.44	< 0.001
Cheek	39.15 ± 5.49	38.49 ± 5.54	0.223	39.22 ± 5.10	38.61± 4.98	0.193
Jowl	43.44 ± 4.56	41.97 ± 4.37	< 0.001	43.18 ± 4.31	42.25 ± 4.05	0.005

## Discussion

To the best of the author's knowledge, this study's findings are the first to systematically analyze and characterize expected differentials among single, duplicate, and triplicate TDC values across multiple facial areas and to investigate the potential impact of patient position on these measurements. The importance of this issue lies in the real-world and perceived time constraints that may limit the use of a potentially useful noninvasive technology for assessing skin hydration, including the water content of the epidermis and dermis, as revealed by TDC measurements. The results indicate that, on average, there are negligible differences between the TDC1 measurement and either the TDC12 measurements or the TDC123 measurements, and that the average differences between measurements made with patients in supine or sitting positions are less than 1%. Thus, the measurements can be made with the subject in a position that is most convenient for the patient and the examiner, without concern about the impact on the TDC-measured value.

When considering positional divergence of TDC values among individual subjects, whether based on single measurements or triplicate averages, the findings indicate that this divergence did not exceed 3% in any subject. This quantitative assessment allows the investigator or clinician to make such measurements and objectively decide whether this 3% threshold is acceptable relative to the time savings associated with using a single TDC measurement.

Comparative absolute TDC data to the present findings of facial TDC are available, but mostly limited to measurements made on females. Nkengne et al. [25] measured C and J areas in 151 women but used a probe with an effective measurement depth of 2.5 mm and reported their values only in graphical form as percentage water, a quantity calculated from the actual TDC value, which is approximately 80% of the percentage water content. Converting their values to TDC yields average TDC values at the cheek and jowl for 31 women aged 20 to 29 to be 42.3 and 35.3, respectively. The lower value they measured at the J area likely reflects the probe's deep penetration, which included the underlying fat at that site. In contrast, measurements in 32 females in the same age range to a depth of 1.5 mm, which includes little or no underlying fat, as was used in the present study, indicated TDC values of 36.8 ± 2.7 and 32.5 ± 3.6 for the F and C, respectively [26]. These values are more consistent with the expected lower TDC values in females than in males, given the greater dermal thickness reported in males [27,28].

The primary limitation of the present study is that the results strictly apply to the young adult male population evaluated. Although it is expected that the results would be largely consistent with other populations that were not male, were older, or had specific skin conditions, this assumption warrants verification in future studies. Although the absolute TDC values have been shown to depend on each of these potential confounders [29,30], there is as yet no evidence to refute the relationship between the TDC value obtained from a single measurement and the average of multiple measurements. 

## Conclusions

TDC measurements of the F, C, and J areas, when made to an effective depth of 1.5 mm, reflect the dermal water at each site with values that are negligibly different when based on the first single measurement compared with the average of multiple TDC measurements. This applies to subjects in either the supine or sitting position. The significance of these results lies in the now-confirmed ability of clinicians or researchers to shorten assessment protocols and to measure facial skin hydration with this method in a position that is comfortable for the patient and examiner, without undue concern about positional effects. 
